# Допубертатная гинекомастия в дебюте синдрома наследственной предрасположенности к опухолям (описание клинических случаев)

**DOI:** 10.14341/probl13239

**Published:** 2023-08-30

**Authors:** М. А. Карева, Л. С. Созаева, И. С. Чугунов, В. А. Петеркова, С. Д. Михалина

**Affiliations:** Национальный медицинский исследовательский центр эндокринологии; Национальный медицинский исследовательский центр эндокринологии; Национальный медицинский исследовательский центр эндокринологии; Национальный медицинский исследовательский центр эндокринологии; Национальный медицинский исследовательский центр эндокринологии

**Keywords:** Синдром Пейтца-Егерса, допубертатная гинекомастия, STK11, Сертоли-клеточные опухоли яичка.

## Abstract

Синдром Пейтца–Егерса (Peutz–Jeghers Syndrome, PJS) относится к синдромам наследственной предрасположенности к опухолям и обусловлен патологическими вариантами гена STK11, приводящими к нарушению синтеза белка серин/треонинкиназы 11, выполняющего роль опухолевого супрессора.Клиническим проявлением синдрома является сочетание гамартоматозного полипоза желудочно-кишечного тракта и характерной кожно-слизистой гиперпигментации. Также для данного заболевания характерен высокий риск развития желудочно-кишечных и внекишечных новообразований, в том числе доброкачественных или злокачественных опухолей репродуктивной системы.Одним из первых проявлений заболевания у пациентов мужского пола может быть допубертатная гинекомастия, связанная с крупноклеточными кальцифицирующими опухолями яичек из клеток Сертоли, экспрессирующими ароматазу. В отличие от пубертатной гинекомастии, допубертатная встречается крайне редко, и в ее основе часто лежат патологические причины. Своевременное установление точного диагноза пациентам с допубертатной гинекомастией, в том числе синдрома Пейтца–Егерса, определяет как тактику ведения гинекомастии, так и протоколы наблюдения за развитием других компонентов заболевания в будущем.В статье представлено описание двух пациентов с допубертатной гинекомастией на фоне синдрома Пейтца–Егерса с разными молекулярно-генетическими дефектами: в одном случае связанной с дупликацией участка гена STK11, в другом — с микроделецией короткого плеча 19 хромосомы, содержащего данный ген.

## Abstract (en)

Peutz-Jeghers Syndrome (Peutz-Jeghers Syndrome, PJS) refers to syndromes of hereditary tumor predisposition and is caused by pathological variants of the STK11 gene, leading to a defect in the synthesis of serine/threonine kinase 11 protein, which acts as a tumor suppressor.Clinical symptoms of the syndrome are combination of hamartomatous polyposis of the gastrointestinal tract and specific skin-mucosal hyperpigmentation. Also, this disease is characterized by a high risk of developing gastrointestinal and extra-intestinal tumors, including benign or malignant tumors of the reproductive system.One of the first signs of the disease in male patients may be prepubertal gynecomastia associated with large-cell calcifying Sertoli cells tumors expressing aromatase. In contrast to from pubertal gynecomastia, prepubertal is extremely rare, and it is often based on pathological causes. Early diagnosis of patients with pre-pubertal gynecomastia, including Peitz-Jaegers syndrome, defines the tactics of gynecomastia management and protocols for monitoring the development of other components of the disease in the future.This article describes two patients with pre-pubertal gynecomastia and Peitz-Jaegers syndrome with different molecular genetic defects: in one case associated with duplication of the STK11 gene site, in the other — with microdeletion of the short arm of chromosome 19 containing this gene.

## АКТУАЛЬНОСТЬ

Гинекомастия — это, как правило, доброкачественная пролиферация ткани молочных желез у представителей мужского пола, возникающая вследствие дисбаланса между действием эстрогенов, стимулирующих рост ткани молочных желез, и блокирующим действием андрогенов. В детском возрасте в подавляющем большинстве случаев гинекомастия имеет физиологический характер и возникает в грудном возрасте и у подростков в период пубертата [1]. Пубертатная гинекомастия возникает, по разным данным, у 20–70% мальчиков-подростков и проходит самостоятельно примерно через 6 мес, в некоторых случаях может персистировать до 1–2 лет, редко она может сохраняться и требовать хирургической коррекции в косметических целях [2]. Возникновение допубертатной гинекомастии (у детей старше года и до начала полового развития) является крайне редким явлением и требует тщательного обследования для исключения патологических причин. Признаками патологической гинекомастии у мальчиков являются [3]:

Причинами допубертатной гинекомастии могут являться [3]:

Опухоли яичек, приводящие к избыточной секреции эстрогенов, могут развиваться самостоятельно, но также могут возникать при наследственных опухолевых синдромах, таких как синдром Пейтца–Егерса и Карни-комплекс [4].

Синдром Пейтца–Егерса (Peutz–Jeghers Syndrome, PJS) — редкое наследственное заболевание, для которого характерно развитие множественных желудочно-кишечных и внекишечных новообразований.

Достоверной информации о распространенности синдрома Пейтца–Егерса в настоящее время нет — частота случаев варьируется от 1 на 8 300 до 1 на 280 000 человек [5].

Впервые обратил внимание на наследственную природу данного синдрома J. Peutz в 1921 г. во время наблюдений за голландской семьей с полипозом желудочно-кишечного тракта. Однако только в 1949 г. H. Jeghers и соавт. описали 10 случаев данного синдрома как отдельное заболевание и выделили характерную триаду: полипоз желудочно-кишечного тракта (ЖКТ), пигментные пятна на коже и слизистых оболочках и наследственный характер заболевания [6].

В основе синдрома лежат мутации гена STK11, который расположен на коротком плече 19 хромосомы и кодирует фермент серин/треонинкиназу 11, относящийся к семейству супрессоров опухолей. В норме данный фермент фосфорилирует 5’АМФ-активируемую протеинкиназу, которая впоследствии запускает каскад реакций, приводящих к подавлению активности регулятора клеточных процессов mTOR, в результате чего происходит остановка роста и пролиферации клеток [7].

Варианты гена (нонсенс- и миссенс-мутации, небольшие делеции/инсерции, большие делеции) приводят к нарушению структуры или сокращению длины кодируемого белка и утрате им киназной активности, что, в свою очередь, увеличивает скорость метаболизма и деление опухолевых клеток, секрецию опухолевых цитокинов и приводит к развитию новообразований [8].

Синдром наследуется по аутосомно-доминантному типу. Риск возникновения заболевания одинаков для мужчин и женщин.

Согласно современным клиническим рекомендациям, установить синдром Пейтца–Егерса можно при наличии любых двух из следующих признаков:

Полипы ЖКТ типа PJS (гамартоматозные полипы PJS-типа) являются особым типом гамартоматозных полипов ЖКТ, которые гистопатологически характеризуются отчетливыми пересекающимися пучками гладких мышц с характерным ветвящимся (ветвистым деревом) видом по всей собственной пластинке [9].

Чаще всего первым признаком заболевания является характерная гиперпигментация слизистых оболочек и кожи, которая обычно проявляется в детском возрасте и предшествует возникновению симптомов, связанных с образованием гамартоматозных полипов ЖКТ. Учитывая риски поражения ЖКТ, такие пациенты требуют тщательного наблюдения у гастроэнтеролога и проведения регулярного обследования с целью своевременного выявления и удаления полипов ЖКТ, что снижает риск злокачественных новообразований и осложнений в виде инвагинации кишечника и кишечной непроходимости. Исходный скрининг ЖКТ должен включать эндоскопию верхних отделов ЖКТ (эзофагогастродуоденоскопию), видеокапсульную эндоскопию и колоноскопию, начиная с восьмилетнего возраста. Последующий интервал скрининга основывается на результатах исходного обследования [10].

Помимо гамартоматозных полипов, у данных пациентов выше риск развития некоторых других опухолей, в том числе злокачественных: рак поджелудочной железы, рак груди, злокачественные опухоли матки и яичников [11].

Однако, помимо перечисленных клинических проявлений, не менее важным является эндокринная составляющая синдрома Пейтца–Егерса — для мальчиков характерны крупноклеточные кальцифицирующие опухоли яичек из клеток Сертоли, неопластические клетки которых экспрессируют ароматазу, что приводит к повышению уровня эстрогенов и развитию гинекомастии, ускорению линейного роста и прогрессии костного возраста [12]; для девочек — эстроген-секретирующие опухоли яичников, которые приводят к развитию преждевременного полового развития по изосексуальному типу [13].

В случае ранней диагностики синдрома и верной тактики ведения таких пациентов продолжительность жизни может достигать 80 лет. Причинами смерти могут быть как последствия полипоза ЖКТ — инвагинация кишечника, желудочно-кишечное кровотечение, так и онкологический процесс (прогноз таких пациентов зависит от вида злокачественного новообразования) [11].

Необходимо отметить, что гинекомастия может быть одним из первых клинических проявлений синдрома, за исключением характерной гиперпигментации слизистых оболочек и кожи, возникающих в раннем возрасте, и являться одной из причин первого обращения пациентов за медицинской помощью [12].

В статье представлено описание двух мальчиков, у которых единственными клиническими проявлениями на момент обследования были характерная кожно-слизистая гиперпигментация и гинекомастия.

## ОПИСАНИЕ СЛУЧАЯ

Пациент С., 4,7 года, поступил в ФГБУ «НМИЦ эндокринологии» МЗ РФ впервые с жалобами на увеличение грудных желез и их болезненность.

Из анамнеза известно, что увеличение грудных желез отмечалось с возраста 3 лет с одновременным появлением темно-синих пятен на коже нижней губы. В возрасте 4 лет мальчик впервые осмотрен детским эндокринологом по месту жительства: рост 116 см, вес — 22 кг, SDS роста +2,5, отмечалась двусторонняя гинекомастия. Половые органы были сформированы правильно, по мужскому типу, стадия полового развития Таннер 1, яички в мошонке по 4 мл.

В гормональном профиле отмечались допубертатные уровни гонадотропинов (лютеинизирующий гормон (ЛГ) — менее 0,09 мМЕ/мл, фолликулостимулирующий гормон (ФСГ) — менее 0,07 мМЕ/мл) и половых гормонов (эстрадиол — менее 37 пмоль/л, тестостерон — 0,17 нмоль/л). По результатам рентгенографии кистей костный возраст несколько опережал паспортный и соответствовал 5,3 годам. При ультразвуковом исследовании органов мошонки отмечалось двустороннее увеличение яичек — объем правого яичка 1,7 см³, объем левого яичка 1,9 см³. Проведена проба с аналогом гонадотропин-рилизинг-гормона — данных за гонадотропинзависимое преждевременное половое развитие не получено (максимальный уровень ЛГ составил 0,29 МЕд/л через 4 ч после начала пробы). С целью уточнения диагноза и решения вопроса о дальнейшей тактике ведения, была рекомендована госпитализация в ФГБУ «НМИЦ эндокринологии».

При осмотре на момент поступления обращали на себя внимание умеренная высокорослость (рост 118,8 см, SDS +2,83, вес 24,3 кг, ИМТ 17,2 кг/м², SDS ИМТ +1,32), а также темно-синие пигментные пятна на коже и слизистой нижней губы, увеличение грудных желез с обеих сторон. Половые органы сформированы правильно по мужскому типу, стадия полового развития — Таннер 1, объем яичек по орхидометру Прадера соответствовал 4 мл с обеих сторон. В гормональном профиле при допубертатных уровнях гонадотропинов (ЛГ 0,21 Ед/л (0–1,5), ФСГ 0,6 Ед/л (0–2)) и тестостерона (0,17 нмоль/л (0,24–0,69)) отмечалось повышение уровня эстрадиола до 84,4 (0–47) пмоль/л. Костный возраст значимо опережал паспортный и соответствовал 7,1 годам. По результатам ультразвукового исследования мошонки отмечалось увеличение размеров яичек: объем правого яичка 1,9 мл, левого яичка — 1,8 мл (рис. 1).

**Figure:**
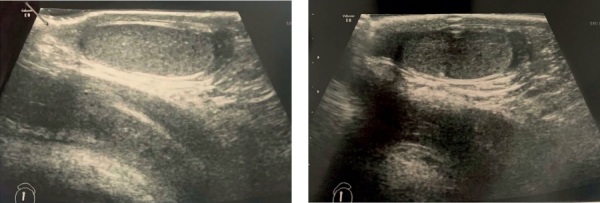
Рисунок 1. УЗИ органов мошонки пациента С. в возрасте 4,7 года.

Из наследственного анамнеза известно, что у матери пациента имеется полипоз ЖКТ (полип двенадцатиперстной кишки, множественные полипы тощей кишки, полип нисходящей ободочной кишки).

Учитывая отягощенный наследственный анамнез по полипозу ЖКТ, наличие темно-синих пятен на коже нижней губы и допубертатную гинекомастию, установлен синдром Пейтца–Егерса. Выполнено молекулярно-генетическое исследование, по результатам которого в 5-м экзоне гена STK11 выявлен гетерозиготный вариант c.646dup:p.S216Ffs*50 (не описан; патогенный (согласно классификации ACMG)). После получения результатов молекулярно-генетического исследования пробанда матери пациента было также проведено исследование гена STK11, по результатам которого выявлен аналогичный генетический вариант.

Учитывая диагноз, наличие у пациента истинной гинекомастии на фоне повышенного уровня эстрадиола при допубертатных гонадотропинах, увеличение объема яичек расценено как гиперплазия клеток Сертоли, экспрессирующих ароматазу.

В связи с наличием гиперэстрогенемии с целью замедления скорости закрытия зон роста и улучшения прогнозируемого роста по решению консилиума пациенту была рекомендована инициация антиэстрогенной терапии блокатором ароматазы III поколения — анастрозолом в дозе 1 мг/сут.

При повторном обследовании пациента через 1 год отмечалась положительная динамика в виде уменьшения выраженности гинекомастии и снижения уровня эстрадиола (53,6 пмоль/л). Однако на этом фоне сохранялись высокие темпы роста и прогрессия костного возраста (по TW20 — 8,3 лет), что, вероятнее всего, было связано с малой продолжительностью терапии анастрозолом. Также при УЗИ органов мошонки выявлены эхографические признаки микрокальцификатов в обоих яичках, которые не визуализировались при первичном обследовании (рис. 2). Было рекомендовано продолжить терапию блокаторами ароматазы. Также ребенку рекомендовано проведение гастро- и колоноскопии в возрасте 8 лет.

**Figure:**
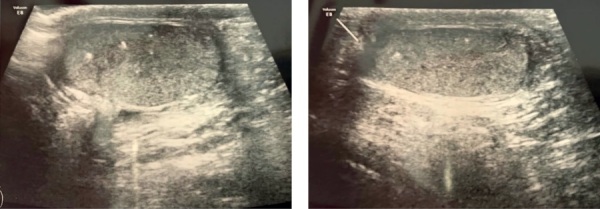
Рисунок 2. УЗИ органов мошонки пациента С. в возрасте 6 лет.

Пациент Ф., 7 лет, госпитализирован в ФГБУ «НМИЦ эндокринологии» МЗ РФ впервые с жалобами на увеличение грудных желез, водянку яичка справа, кальцинаты в яичках по результатам УЗИ.

Из анамнеза известно, что в возрасте 2 мес в связи с наличием стигм дизэмбриогенеза (эмбриональная грыжа, дисгенезия мозолистого тела по данным МРТ головного мозга) выполнен хромосомный микроматричный анализ, по данным которого выявлена микроделеция короткого плеча 19 хромосомы с позиции 352288 до позиции 1434508, в зону которой также попадает ген STK11.

С 5 лет у мальчика отмечалось увеличение грудных желез, с выраженной прогрессией после 6,5 лет. При обследовании по месту жительства в возрасте 6,5 лет в гормональном профиле на фоне допубертатных гонадотропинов (ЛГ 0,12 мМЕд/мл, ФСГ 0,08 мМЕд/мл) и тестостерона (0,14 нмоль/л) отмечалось повышение уровня эстрадиола (44,1 пмоль/л). По результатам рентгенографии кистей костный возраст опережал паспортный и соответствовал 7 годам. При ультразвуковом исследовании органов мошонки отмечались признаки водянки правого яичка, множественные кальцинаты, преимущественно в правом яичке. Была проведена проба с аналогом гонадотропин-рилизинг-гормона — данных за гонадотропинзависимое преждевременное половое развитие не получено (максимальный уровень ЛГ составил 0.53 мМЕд/мл).

С целью уточнения диагноза и решения вопроса о дальнейшей тактике ведения, была рекомендована госпитализация в ФГБУ «НМИЦ эндокринологии».

При осмотре на момент поступления: рост 120,8 см, SDS роста +0,09, вес: 21,5 кг, SDS ИМТ -0,62. Отмечались темно-синие пигментные пятна на носу, щеках и губах, увеличение грудных желез с обеих сторон, рост остистых волос на спине, готическое небо, широкая щель между 1-м и 2-м пальцами. Половые органы были сформированы правильно по мужскому типу, стадия полового развития по Таннер 1, яички в мошонке, объем яичек по орхидометру Прадера соответствовал справа 3 мл, слева 4 мл. Также справа были признаки водянки.

В гормональном профиле отмечалось повышение уровня эстрадиола — 128,48 пмоль/л (0–47) при допубертатных уровнях ЛГ — 0,216 Ед/л (0–1,5), ФСГ — 0,66 Ед/л (0–2), тестостерона 0,176 нмоль/л (0,24–0,69). Костный возраст несколько опережал паспортный и соответствовал 8,6 годам. По результатам ультразвукового исследования мошонки объем правого яичка 1,5 мл, левого яичка — 1,5 мл, эхографические признаки микрокальцификатов в обоих яичках, очаговых изменений в правом яичке, водянки оболочек правого яичка (рис. 3).

**Figure:**
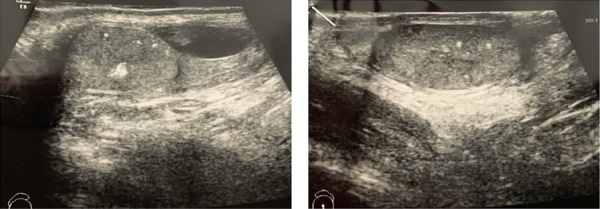
Рисунок 3. УЗИ органов мошонки пациента Ф.

Синдром Пейтца–Егерса у данного пациента не вызывал сомнений в связи с наличием типичных клинических проявлений и результатов молекулярно-генетического исследования.

Наличие у пациента стигм эмбриогенеза объясняется микроделецией с выпадением не только гена STK11, но также генов: THEG, C2CD4C, SHC2, MADCAM1, CDC34, GZMM, BSG, HCN2, POLRMT, FGF22, FSTL3, PALM, AZU1, PRTN3, ELANE, CFD, MED16, KISS1R, ARID3A, GRIN3B, C19orf66, CNN2, ABCA7, HMHA1, POLR2E, GPX4, ATP5D, MIND, CIRBP, EFNA2, NDUFS7, GAMT, DAZAP1. Учитывая делецию данных генов и возможное развитие пороков внутренних органов, пациенту дополнительно был проведен скрининг возможных аномалий, по результатам которого данных за патологию сердца, почек не получено.

Учитывая наличие гиперэстрогенемии, с патогенетической целью по решению консилиума пациенту была рекомендована инициация антиэстрогенной терапии блокатором ароматазы III поколения — анастрозолом в дозе 1 мг/сут. При обследовании пациента через 1 год отмечалась положительная динамика в виде снижения уровня эстрадиола до 63 пмоль/л и отсутствия прогрессии костного возраста. Пациент был направлен в специализированное гастроэнтерологическое отделение для проведения капсульной колоноскопии и гастроэзофагодуоденоскопии.

## ОБСУЖДЕНИЕ

Допубертатная гинекомастия встречается крайне редко, в отличие от неонатальной и пубертатной гинекомастии, которые развиваются более чем у половины мальчиков. Причины препубертатной гинекомастии можно разделить на спорадические опухоли и наследственные синдромы.

К спорадическим опухолям относятся ХГЧ-секретирующие опухоли как гонадной, так и внегонадной локализации, феминизирующие опухоли яичек (из клеток Лейдига и Сертоли) и надпочечников. К наследственным синдромам относятся: синдром Пейтца–Егерса, Карни-комплекс, синдром избытка ароматазы [14].

Наиболее частым эндокринным проявлением при синдроме Пейтца-Егерса у мальчиков являются крупноклеточные кальцифицирующие опухоли яичек из клеток Сертоли, неопластические клетки которых экспрессируют ароматазу, что приводит к гинекомастии, ускорению линейного роста и прогрессии костного возраста. При ультразвуковой диагностике чаще всего отмечается двустороннее увеличение объема яичек с визуализацией микрокальцинатов в виде «рождественской елки», однако, по данным литературы, также может отмечаться двустороннее увеличение размера яичек без микрокальцинатов [4].

В данной статье были представлены два клинических случая пациентов с синдромом Пейтца–Егерса. У обоих пациентов были выявлены компоненты данного синдрома — характерная кожно-слизистая гиперпигментация и изменения яичек, наиболее вероятно соответствующие Сертоли-клеточным опухолям яичек, а также проявления, связанные с гиперэстрогенемией — гинекомастия, ускорение темпов линейного роста и опережение костного возраста.

Надо отметить, что пациенты отличаются фенотипически, что связано не только с возможным полиморфизмом проявлений этого заболевания, но и с тем, что у первого пациента была мутация только в гене STK11, а у второго имеет место микроделеционный синдром в связи с делецией гена STK11 и еще 33 генов, попадающих в зону делеции.

В связи с неясным злокачественным потенциалом данных опухолей единой тактики ведения пациентов, имеющих Сертоли-клеточные опухоли, нет. В литературе описаны случаи, где отдавали предпочтение двусторонней орхиэктомии, принимая во внимание возможный инвазивный рост данных опухолей. Однако, учитывая низкий риск злокачественной трансформации опухолей полового канатика, в случае диагностики кальцифицирующих образований яичка в допубертатном возрасте в настоящее время отдают предпочтение консервативной тактике ведения с целью сохранения сперматогенеза и возможности развития самостоятельного полового созревания. В качестве консервативного лечения используют блокаторы ароматазы III поколения с целью снижения уровня эстрогенов и тщательное наблюдение за размерами и характеристиками опухолей яичек при помощи УЗИ [12].

При лечении наших пациентов также была выбрана консервативная тактика ведения — обоим мальчикам назначена терапия ингибиторами ароматазы с положительным эффектом в виде снижения уровня эстрогенов. Пациенты продолжают наблюдаться эндокринологами, онкологами и гастроэнтерологами.

Учитывая то, что гинекомастия при данном синдроме является одним из первых значимых компонентов, такие пациенты в первую очередь могут обращаться к педиатрам и эндокринологам. Установление точного диагноза и причины гинекомастии крайне важно для детей с синдромом Пейтца–Егерса, так как позволит не только выбрать адекватную тактику лечения данного проявления, но и своевременно начать скрининговое обследование, принимая во внимание возможность развития у пациентов множественных опухолей других локализаций, тем самым улучшив качество и продолжительность их жизни.

## ЗАКЛЮЧЕНИЕ

Синдром Пейтца–Егерса — редкое наследственное заболевание, которое можно заподозрить на основании отягощенного наследственного анамнеза и характерных клинических проявлений (гиперпигментация слизистых оболочек и кожи, полипоз ЖКТ). Одним из ранних компонентов синдрома являются крупноклеточные кальцифицирующие опухоли яичек, экспрессирующие ароматазу, которые могут возникать в детстве, что делает необходимым включение синдрома Пейтца–Егерса в спектр причин для дифференциальной диагностики у мальчиков с гинекомастией в допубертатном возрасте. Установление диагноза необходимо для комплексного междисциплинарного подхода к данной группе пациентов, а также для проведения медико-генетического консультирования семьи и выбора оптимальной программы наблюдения за пациентами.

## ДОПОЛНИТЕЛЬНАЯ ИНФОРМАЦИЯ

Источники финансирования. Молекулярно-генетическое исследование выполнено при финансовой поддержке фонда поддержки и развития филантропии «КАФ».

Конфликт интересов. Все авторы декларируют отсутствие явных и потенциальных конфликтов интересов, связанных с публикацией настоящей статьи.

Участие авторов. Карева М.А., Созаева Л.С., Чугунов И.С., Петеркова В.А., Михалина С.Д. — концепция и дизайн исследования, сбор материала, анализ полученных данных, написание текста. Все авторы внесли значимый вклад в подготовку статьи, прочли и одобрили финальную версию статьи перед публикацией, выразили согласие нести ответственность за все аспекты работы, подразумевающую надлежащее изучение и решение вопросов, связанных с точностью или добросовестностью любой части работы.

Согласие пациента. Законные представители пациентов подписали информированное согласие на публикацию персональной медицинской информации в журнале «Проблемы эндокринологии» в обезличенной форме.

## References

[cit1] Patrick Mahoney C. (2016). Adolescent Gynecomastia: Differential Diagnosis and Management. Pediatric Clinics of North America.

[cit2] Mieritz Mikkel G., Rakêt Lars L., Hagen Casper P., Nielsen John E., Talman Maj-Lis M., Petersen Jørgen H., Sommer Stefan H., Main Katharina M., Jørgensen Niels, Juul Anders (2015). A Longitudinal Study of Growth, Sex Steroids, and IGF-1 in Boys With Physiological Gynecomastia. The Journal of Clinical Endocrinology & Metabolism.

[cit3] Ma Nina S, Geffner Mitchell E (2009). Gynecomastia in prepubertal and pubertal men. Current Opinion in Pediatrics.

[cit4] Gourgari Evgenia, Saloustros Emmanouil, Stratakis Constantine A. (2012). Large-cell calcifying Sertoli cell tumors of the testes in pediatrics. Current Opinion in Pediatrics.

[cit5] Lindor N. M., McMaster M. L., Lindor C. J., Greene M. H. (2008). Concise Handbook of Familial Cancer Susceptibility Syndromes - Second Edition. JNCI Monographs.

[cit6] Jeghers Harold, McKusick Victor A., Katz Kermit H. (2010). Generalized Intestinal Polyposis and Melanin Spots of the Oral Mucosa, Lips and Digits. New England Journal of Medicine.

[cit7] Mihaylova MM, Shaw RJ. The AMPK signalling pathway coordinates cell growth, autophagy and metabolism. Nat Cell Biol. 2011;13(9):1016-1023. doi: https://doi.org/10.1038/ncb2329 21892142 PMC3249400

[cit8] Schumacher V (2005). STK11 genotyping and cancer risk in Peutz-Jeghers syndrome. Journal of Medical Genetics.

[cit9] Beggs A. D., Latchford A. R., Vasen H. F. A., Moslein G., Alonso A., Aretz S., Bertario L., Blanco I., Bulow S., Burn J., Capella G., Colas C., Friedl W., Moller P., Hes F. J., Jarvinen H., Mecklin J.-P., Nagengast F. M., Parc Y., Phillips R. K. S., Hyer W., Ponz de Leon M., Renkonen-Sinisalo L., Sampson J. R., Stormorken A., Tejpar S., Thomas H. J. W., Wijnen J. T., Clark S. K., Hodgson S. V. (2010). Peutz-Jeghers syndrome: a systematic review and recommendations for management. Gut.

[cit10] Boland C. Richard, Idos Gregory E., Durno Carol, Giardiello Francis M., Anderson Joseph C., Burke Carol A., Dominitz Jason A., Gross Seth, Gupta Samir, Jacobson Brian C., Patel Swati G., Shaukat Aasma, Syngal Sapna, Robertson Douglas J. (2022). Diagnosis and Management of Cancer Risk in the Gastrointestinal Hamartomatous Polyposis Syndromes: Recommendations From the US Multi-Society Task Force on Colorectal Cancer. Gastroenterology.

[cit11] van Lier M G F, Wagner A, Mathus-Vliegen E M H, Kuipers E J, Steyerberg E W, van Leerdam M E (2010). High Cancer Risk in Peutz–Jeghers Syndrome: A Systematic Review and Surveillance Recommendations. American Journal of Gastroenterology.

[cit12] Lefevre Hervé, Bouvattier Claire, Lahlou Najiba, Adamsbaum Catherine, Bougnères Pierre, Carel Jean-Claude (2006). Prepubertal gynecomastia in Peutz-Jeghers syndrome: incomplete penetrance in a familial case and management with an aromatase inhibitor. European Journal of Endocrinology.

[cit13] Young Robert H., Dickersin G. Richard, Scully Robert E. (2006). A distinctive ovarian sex cord-stromal tumor causing sexual precocity in the Peutz-Jeghers syndrome*. The American Journal of Surgical Pathology.

[cit14] Laimon Wafaa, El-Hawary Amany, Aboelenin Hadil, Elzohiri Mohamed, Abdelmaksoud Sherif, Megahed Nirmeen, Salem Nanees (2020). Prepubertal gynecomastia is not always idiopathic: case series and review of the literature. European Journal of Pediatrics.

